# Unexpected effects of urban food activism on community and human wellbeing

**DOI:** 10.1080/13549839.2023.2298675

**Published:** 2024-01-12

**Authors:** Jennifer Wardle, John McKenzie, Martin Barker, David F.R.P. Burslem, Donald Gray

**Affiliations:** a School of Biological Sciences, University of Aberdeen, Aberdeen, UK; b Rowett Institute, Aberdeen, UK; c School of Education, University of Aberdeen, Aberdeen, UK

**Keywords:** Volunteer burnout, community gardens, coordinator wellbeing, urban agriculture

## Abstract

Participation in urban agriculture conducted through community gardens and allotments is known for its benefits to physical and mental health. Due to the recognition of these benefits, which include reduction of stress, depression and anxiety, such participation is increasingly being prescribed as a non-medical health intervention. Community gardens have the added advantage of immersion into a community, without the often-long waiting lists and level of commitment involved in allotment tenancies. What has not been explored is the demanding nature of the commitment required by volunteer coordinators, and ironically, the negative effects it can have on their wellbeing. In a study of food activism in Aberdeen, UK, we conducted 21 semi-structured interviews with participants from a range of bodies involved in the city’s food growing projects. From the spectrum of food growers, we found that volunteer coordinators of community gardens experienced the greatest burdens on their time and wellbeing, with their demanding multi-functional roles leading to fatigue and feelings of over-commitment. Other problems encountered by community gardeners were over-reliance on grant funding and the disproportionate impacts of COVID closures on vulnerable groups. Policy interventions are required to reduce dependency on competitive grant funding and to support both coordinators and the long-term sustainability of community gardens.

## 1 Introduction

The positive effects of gardening, community food activism, social-connectivity, and being outdoors include reductions in stress, depression and anxiety and improvements in health, self-esteem, sleep patterns and overall wellbeing (Bu et al. 2021; Glover 2021; van den Berg et al. 2010; Wood, Pretty, and Griffin 2016). Community food growing can be a means of instigating collective action (Nam and Dempsey 2018), facilitating the enhancement of employability skills (Ramsden 2021; St Clair et al. 2018), and developing social and cultural resilience through creating space for communication, knowledge exchange and shared decision-making (Cumbers et al. 2018; King 2008; St Clair et al. 2018).

Much of the literature on urban food growing focuses on its benefits, with little attention paid to its challenges. Authors that do address negative aspects, particularly for community gardens, tend to focus on neoliberalism and political agendas causing unequal access to food, lack of social security, and gentrification of neighbourhoods (Haase et al. 2017; McClintock 2014; Sonnino and Hanmer 2016). Others point out that these groups are predominately white and middle-class in themselves (Ramsden 2021; St Clair et al. 2018), or include multi-ethnic groups under white, middle-class leadership (Furness and Gallaher 2018; Glover 2021), which can exacerbate cultural exclusion (McVey, Nash, and Stansbie 2018).

Despite the growing body of literature on urban agriculture and community gardens, there has been little mention of the constraints to their long-term sustainability or the negative impacts on health and wellbeing that may arise from such ventures. The burden of these responsibilities is arguably a threat to the longevity of the gardens and related organisations. In this paper we aim to examine the positive and negative impacts of involvement in urban food growing in general, and how that participant experience was affected by the COVID lockdown period of 2020/21. In particular, we explore the challenges to mental health and wellbeing associated with volunteers with roles of responsibility in the movement.

Throughout history, crises such as war and economic difficulties have initiated a spate of urban food growing initiatives, often facilitated by allotments (Miller 2015; Nam and Dempsey 2018; RHS 2021; Schoen et al. 2021). The most recent crisis was the COVID pandemic and the measures taken to control it (Joshi and Wende 2022; Schoen et al. 2021). According to the Association for Public Service Excellence (APSE 2021), 94% of responsive councils experienced a pandemic-related increase in demand for allotments, as well as an increase in the frequency of visits by allotment tenants to their plots. Allotments holders were seen to have more time available for gardening due to time saved in working from home or through furlough (Bu et al. 2021; Schoen et al. 2021) and may have been incentivised by the significant disruptions to global food systems (Court, Kelly, and Hardman 2022). In the initial UK strict lockdown period, March-June 2020 (Institute of Government Analysis 2022), one of the few legally accepted reasons for being outdoors away from home was to maintain an allotment (Mason 2020). Due to a multitude of people experiencing the same circumstances, social contact within allotments was reported to increase as more people were present and could keep at a safe distance within their own plots (Schoen et al. 2021).

In contrast, the COVID pandemic had opposite effects on many community gardens in the UK, with usage decreasing or ceasing due to legal restrictions on group gatherings and some individual’s own fear of group environments (Schoen et al. 2021). In some cases, knock-on effects were felt when regulations eased, as younger members became demotivated by the continued absence of older, more experienced growers on whom they relied for guidance, with travel remaining a deterrent for those relying on public transport (Schoen et al. 2021).

Community gardens are, for the most part, a recent innovation in urban gardening; Web of Knowledge indexed research articles including “community garden*” in the abstract increased from 9 in 2010 to a peak of 121 in 2020. The recent rise has attracted attention beyond those with horticultural interests, with a current trend of social prescribing to community gardens as a non-medical intervention for improving public health (McGuire, Morris, and Pollard 2022; Moore and Thew 2023). In Scotland, urban agriculture, and particularly community gardening, has been promoted by The Community Empowerment (Scotland) Act 2015, which requires local authorities to create food growing strategies, identify potential sites and explore how community growing can be implemented, particularly in economic deprivation zones (Scottish Government 2017). The UK Localism Act 2011 has also facilitated local communities to claim land for food gardens but is less explicit regarding such activities.

Community gardening schemes are generally staffed by volunteers. Volunteer work often leads to positive personal outcomes in addition to the societal benefits it frequently aims to achieve. The advantages of volunteering are similar to those associated with gardening and being outdoors, including improved physical and mental health, self-esteem, social connectedness and decreased feelings of stress, depression and morbidity (Morse et al. 2022). Less well acknowledged is the fact that it can also lead to negative personal outcomes through the onset of burnout syndrome (Kulik 2007; Morse et al. 2022). Burnout syndrome is a feeling of emotional distress and disillusionment about work caused by a discrepancy between expectations and reality (Mealer et al. 2016). To date, most studies on burnout syndrome have been linked to health-care workers who are viewed as having a particularly high vulnerability (Chirico et al. 2021), although it can occur across all sectors. Burnout syndrome is still poorly understood in volunteers from all types of organisations (Morse et al. 2022). It is typically attributed to the three main factors identified as exhaustion (having nothing left to offer), cynicism/depersonalisation (feeling distant towards the work and colleagues) and inefficacy (a feeling of underperforming) (Chirico et al. 2021).

Schoneboom (2018) examined the interactions between allotment maintenance and employment workload on the wellbeing of those in full-time employment. This was based on the recognition of the increasing encroachment of work into personal life, which arguably has become considerably stronger since the normalisation of working from home during lockdown. A key finding was that people reacted to work pressures by busying themselves with allotment tasks to prevent themselves from succumbing to additional work hours (Schoneboom 2018). However, there has been no equivalent research on the effects of volunteers who coordinate community gardens alongside other employment. Those with organisational responsibilities in community gardens have multiple roles, including the design and promotion of activities, securing funding and other resources, and acting as the gatekeeper (McGuire, Morris, and Pollard 2022). Until McGuire, Morris, and Pollard’s (2022) publication, there was a distinct lack of narrative about the wide-ranging roles and responsibilities associated with garden coordinators. In contrast to the hands-on, earthy tasks involved in allotment maintenance, these largely administrative tasks could potentially be viewed as an extension to working life rather than an antidote to it.

The aim of this paper is to highlight the wide-ranging tasks and skillsets required of coordinators, and the unexpected challenges encountered when pursuing goals to promote the benefits of food growing. We also explore how agency within communities can be used to mitigate some of these challenges.

## 2 Methods

This study focussed on urban food growing in the city of Aberdeen, UK, which has an increasing number of community gardens and allotment spaces and received the Silver Sustainable Food Cities Award in 2022 (CFINE 2022). Aberdeen responded to the Community Empowerment (Scotland) Act by establishing the Granite City Growing food strategy (Aberdeen City Council 2020b) and currently has 23 community gardens and 23 allotment sites listed on its map, with many of the gardens established in, or since, 2019 (Aberdeen City Council 2020a).

Twenty-one semi-structured qualitative interviews of approximately 45 minutes each were conducted throughout October–December 2021 to seek a wide range of perspectives on food activism across Aberdeen. By this point a degree of normality had returned after the COVID-19 lockdown, but precautionary measures were still in place. Initial leads were taken through contact details given on an online map that lists allotment sites, community gardens and educational bodies involved in growing activities (Aberdeen City Council 2020a), as well as related websites and social media. There-after, a snowballing technique was used where interviewees recommended key people within the local growing community, as well as disseminating information about the project around their local groups for willing people to contact us.

**Figure 1:**
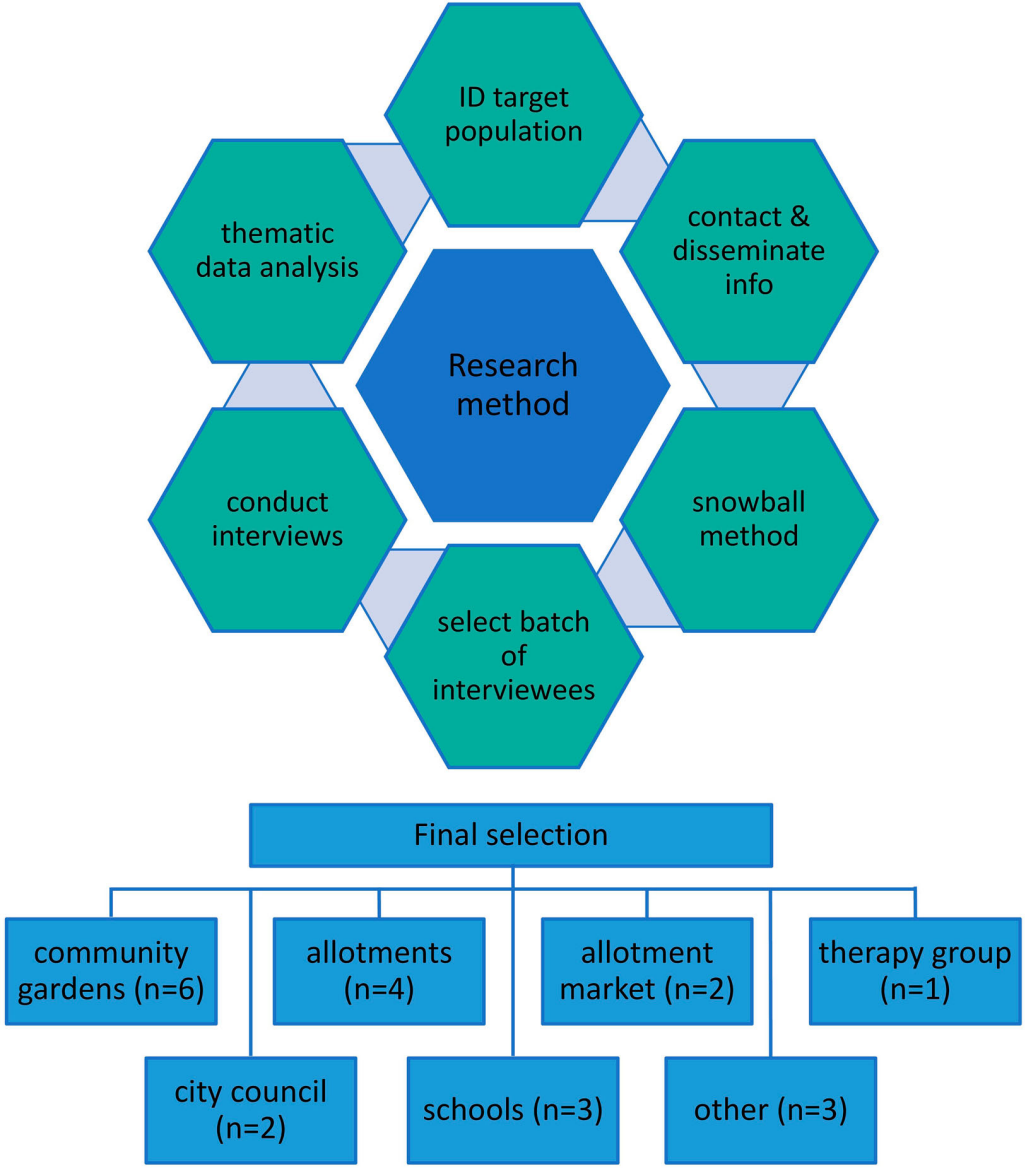
Research strategy and participant group affiliations.

Respondents were selected due to their involvement with relevant organisations and bodies shown in Figure 1, where “other" relates to an initiative promoting food growing education and activities, and a charitable community food network. Most participants were directly involved in urban food growing, while a few had over-arching coordination or management roles, e.g. local council. However, several participants had more than one affiliation type, giving them a broad perspective on the different movements. Although we engaged one community garden volunteer who had few responsibilities, most were coordinators who had greater knowledge of the history and functioning of the gardens. Due to difficulties encountered in engaging schools within Aberdeen city, participants were invited from schools in the wider Aberdeenshire local authority area. In order to gain a greater understanding around the movements, available websites and social media pages were visited before interviews took place.

Interview guides contained prompts relating to participant role, motivations and benefits, group aims, challenges, food production, environment, community and inclusivity. COVID was not explicitly included in the questions, but frequently emerged as an important participantled theme due to the timing of the study and the strong impact it had on people’s lives. Two of the 21 interviews included two interviewees, meaning a total of 23 participants were involved. Participants chose whether interviews were conducted online or face-to-face, with eight opting for the latter. Of the 23 respondents, 11 were male and 12 were female. Based on information freely given in interviews, ages ranged from twenties to seventies. Gender neutral pseudonyms have been used throughout for purposes of anonymity, and the names of organisations are omitted.

Ethical approval was obtained from the University of Aberdeen’s School of Education before participants were contacted. Interviews were audio recorded and transcribed in full by a university approved third party. If interviewees made relevant comments before or after recording, the interviewer took notes which were validated by emailing them to participants to confirm the text was representative. Transcriptions were checked against recorded interviews before being coded in NVIVO Pro v12, using iterative and incremental methods. The thematic analysis was iterative inductive, drawing on both empirically derived themes emerging from the data and informed by those in the existing literature (O’Reilly 2005). Coding was undertaken between interviews, with cross checks on coding conducted by other team members. Analysis drew on grounded theory methods (Bryant 2017), though the emphasis was on novel experiential insights rather than generating theory. After 21 interviews, the same themes were frequently reemerging, indicating that data saturation may have been reached. Themes and frequently occurring words were also visualised and explored in the software to assist with checks on thematic coding.

## 3 Results and discussion

### 3.1 Effects of the COVID pandemic on urban gardening communities

#### 3.1.1 Finding respite during the COVID lockdown

The COVID pandemic provided opportunities but simultaneously created challenges for urban gardening communities. As with the majority of other UK council areas (APSE 2021), an Aberdeen City Council official confirmed “our allotment waiting list has absolutely shot up”, highlighting the need felt by the population to spend more time outside resulting from enforced indoor confinement. Corroborating the findings by Schoen et al. (2021) about the benefits of social interactions during lockdown, an allotment spokesperson talked of appreciating visits to the site for that reason:

It was very good when COVID rules were more strict because you were allowed to go up to your allotment and obviously it was all socially distanced and you could still have a chat with people because they would be like five, ten metres away.

This opportunity was not extended to most community gardeners, but coordinators, who had access rights, enjoyed the benefits of having a restorative outdoor space to be in. Stef described how going to the garden improved their mental health.

I really suffered from anxiety and panic attacks and the only thing that stopped with a panic it was take care of my plants. So this is something that slowed me down and relaxed me, and this is the same that happened when we had the first lockdown, because you couldn’t do anything. I live alone; I was alone all day and the only thing I could do is actually go to the garden and just take care of the plants.

These sentiments are consistent with other findings that gardening for 30 minutes a day during lockdown effectively reduced anxiety and depression (Bu et al. 2021) and provided stress relief (Joshi and Wende 2022). Another coordinator was also appreciative of the green space during lockdown, with furlough allowing time to address the workload involved in setting up the garden while not under financial stress.

#### 3.1.2 Negative impacts on gardening communities caused by the COVID pandemic

Community gardens can be more effective at engaging a wider demographic than allotments, as they offer no-commitment opportunities for residents who would not have contemplated obtaining their own allotment plot due to lack of time, skill, interest or ability to commit to one. One participant demonstrated this, telling us they started volunteering because a local garden emerged, seeing it as a good opportunity for someone who quite liked gardening but did not know much about it. The COVID pandemic, however, showed that community gardens are more susceptible to disruptions than allotments, potentially making them less resilient as long-term ventures. While allotments thrived during lockdown, many UK community gardens were effectively shut down due to the enforcement of laws forbidding social mixing. Although some found means to stay open with safety measures in place to prevent socialising (Schoen and Blythe 2020), a recent study found that over a third of members reported feelings of negativity associated with isolation in the garden, restricted access and the transition from communal to individual activities (Joshi and Wende 2022). In Aberdeen, essential activities were often limited to coordinators who held the keys and responsibilities. This was badly timed for Aberdeen’s newly established garden communities which had just begun to gain momentum, causing concerns for new and inexperienced coordinators about safe-guarding. “COVID was a bit of a challenge because we were just ready to start getting people on site and involved in things and kind of thought, I don’t know how to do that safely now.”

Although most participants were disadvantaged through lockdown, vulnerable groups were hit harder by the cessation of gardening group activities (Ramsden 2021). Apart from the potential increase in inactivity and isolation, the term “gardener” is an identity (McGuire, Morris, and Pollard 2022) and can be used to counter negative collective identities (Jackson 2018). In Aberdeen, “gardener” was a refreshing label for therapy group members who were otherwise stuck with derogatory labels, with the leader seeing that previous garden activities had led to positive interactions with people from outside the group. In addition to the discontinuation of gardening, social activities associated with therapy groups, such as cooking and eating together, were also stopped, leading to further isolation. An allotment outreach coordinator lamented on how the therapeutic and vulnerable groups had abruptly ceased their visits to community plots, which had still not been resumed by the time of the interview in Nov 2021. The concern for vulnerable people missing out on therapeutic garden sessions was shared by policy makers in the study by Schoen et al. (2021) who had a notion that the connections would be difficult to re-establish.

### 3.2 Strains on human wellbeing from coordinating urban gardening activities

I think if you’re wanting to start up any sort of project or community space that involves other people, …. it can put a massive strain on your personal life, your own time and your balance and to how to keep your head level. I think anyone that’s willing to do it really needs to get a lot of help and support

Most participants had experienced some degree of challenge, with the load often falling most heavily on those in volunteer coordinator roles. The extent and nature of the obstacles faced were dependent on the structure of the organisation or institution involved, with volunteer coordinators, including those for therapy groups, activist groups and allotment community plots, all appearing to feel the strain. Despite expressing a labour-of-love attitude towards their role, the recurring theme of work overload was evident. Their motivations for creating communities focused on the value they placed on sustainable food, improving greenspaces and reducing social isolation, but their enthusiasm was dampened by factors including the extent of the bureaucracy involved, structural restrictions, and the time and effort involved in striving towards their goals. This was particularly apparent in those of working age who took on their roles alongside paid work commitments, but these frustrations were also expressed by retired workers. Ultimately, these strains, combined with the volatility of funding, discussed below, appear to be the biggest threats to the longevity of the community groups.

#### 3.2.1 Challenges of establishment

it’s full-time roles that you end up doing when you’re developing a space.

The biggest obstacles reported by those setting up new gardens included the complex processes of obtaining land use permissions and forming constitutionalised committees to meet funding eligibility criteria. This stream of tasks entailed finding the landowner, obtaining land rights, recruiting committee members with a shared vision, agreeing a constitution and setting up group bank accounts and insurance. A support worker confirmed that these were key barriers that groups needed help to overcome, with such struggles being consistent with findings by Court, Kelly, and Hardman (2022) in Greater Manchester. In one case, substantial time and effort was spent following official guidance on procedures to obtain land that subsequently turned out to be incorrect, leaving the prospective coordinator tired and frustrated at inception point.Meanwhile, another coordinator who was a confident gardener since childhood, felt an overwhelming sense of insecurity after initiating the process of setting up a community garden, with confidence replaced by thoughts of “Oh, god, I don’t know what I’m doing or where I’m starting”. Comparable sentiments were expressed by others who took similar leaps. Work that was initiated by a passion for gardening and community building therefore took on unexpected dimensions, including feelings of powerlessness and self-doubt.

Such challenges can diminish the desire to form garden communities, ultimately being more navigable by those with the education, affluence, professional skills and social resources necessary to embark on the process. However, these attributes are less likely to be possessed by those living in deprived areas that would gain the greatest benefit, highlighting the uneven playing field of who survives in the establishment of community gardens (McClintock 2014; McVey, Nash, and Stansbie 2018). A volunteer who was instrumental in connecting and skill-building within Aberdeen’s food growing communities expressed frustration at the feeling that “there’s a lot of people who’d quite like to do things and just can’t because they don’t know where to start.”

#### 3.2.2 Early days and continuity

Groups that survived the establishment process faced further challenges in maintaining continuity. Ongoing demands to promote the site, inform the local community of its existence and purpose, recruit and coordinate volunteers, organise events, and write grant applications left many coordinators citing fatigue or “taking on too much” as a major challenge. Again, a suite of often unfamiliar hard and soft skills (e.g. website-building, team-building and resourcefulness) was required to achieve these objectives. The diverse nature of the skills and tasks required is rarely captured by the literature (McGuire, Morris, and Pollard 2022) and remains largely uninvestigated. The bureaucratic, outreach and organisational responsibilities were often an addition to the demands of paid work or study obligations, with a new coordinator noticing how the return to work after furlough impacted on their time and energy available for garden responsibilities. The work-life balance for community garden leaders apparently contrasts with those of allotment holders assessed by Schoneboom (2018). While Schoneboom (2018) argued that the demands of allotments are constructive in forcing people to make leisure time to counter the intensification and extensification of work into personal time, in this case, the type of tasks involved were an addition to work rather than a counter to it. Conversely, a purely hands-on garden volunteer, claimed that their only challenge was to avoid back pain, but felt empathetic towards those with more responsibility who inevitably did, “Oh, I’m sure they’ve got challenges every day if you asked Les and the others. Yes, yes”.

The workload was arguably intensified by the aspiration to do the tasks well, acknowledging that people wanted to feel warm connections and have useful tasks to work on. Coordination of gardening volunteers, while fundamental to organisational objectives, proved difficult within some structures. Volunteers were encouraged to give advance notice of attendance, but often arrived without prior notice. Coordinators accepted that volunteers should enjoy sessions and not feel bound by commitments, but conceded it was taxing to find people meaningful tasks on the spur of the moment. Ironically, they could not apply the logic of being unbound to themselves, mainly due to feelings that it would not go well without them and concerns over safe-guarding. Sasha, a therapy group leader who balanced two part-time jobs with variable hours, found this aspect of the role particularly stressful. Past experience had made it apparent that finding suitable cover from someone with Protecting Vulnerable Groups training, basic knowledge of gardening and who was comfortable with the client group was a major challenge. Nonetheless, they felt that any days off would impact negatively on the continuity of the group and should be avoided, creating struggles and concerns over practicalities and organisation. In addition to the work on site, Sasha regularly texted all members to confirm sessions, dealt with referrals from other agencies, held fundraisers, attended meetings, and was active in getting the group involved in wider projects such as Britain in Bloom, which incorporated a surprising amount of administration work.

Similar pressures were felt by Noah who ran their group website and coordinated multiple school and therapy groups on an allotment site. Having a strong background in the relevant skillsets, Noah felt adept at managing the community plots until vulnerable groups became involved, with the new situation creating a sense of doubt and inadequacy. After seeking training, which proved elusive, Noah was merely given a proverbial pat on the back and told to get on with it. Both leaders had concerns about the continuity of their respective groups if they stood down, with Noah stating, “one thing I’m not doing, is I’m not giving up the volunteer squad, I’m still going to coordinate that because if I was just to abruptly say I was going I think it would fall to bits.”

In most cases, no conflict was raised about the usage of the space, but for one group, verbal abuse from neighbours induced stress. This was attributed to a lack of communication with residents about usage of the space and locking the gate, which irritated local dog walkers and others using it as a throughfare. Backing up the sentiments of other coordinators, this highlights that effective communication and outreach is not an optional nicety, but an essential element for a community space which requires ongoing effort. Issues of exclusivity and detachment from local communities are often brought up in studies of community growing spaces (Ramsden 2021; St Clair et al. 2018; Traill 2021), with Traill (2021) labelling the exclusion of other groups as rejectionist practice. The collective ownership of community gardens is viewed as essential for unlocking their benefits (McGuire, Morris, and Pollard 2022), which is corroborated by the successes of those who possessed this sense of ownership and the frustration of those who lacked it.

#### 3.2.3 Funding pressures

The recent expansion of community gardens generally occurred earlier in the US than the UK allowing lessons to be learned from the experience. McClintock (2014) reports that funding can be uncertain and dependent on funders’ preferences, with community gardens seen to have a well-funded honeymoon period of just 4–5 years. In Aberdeen, most gardens started around 2019 and are still within the honeymoon period, therefore funding opportunities appeared to be adequate for most and even plentiful for schools, with one staff member claiming, “You can get funding for anything if you are trying to grow”. However, the sense of achievement at the increasing number of community growing spaces was dampened for some by the acknowledgement that not all of the initiatives could continue to be supported by charities.

Several participants expressed discomfort about the reliance on external funding to purchase tools, compost, materials, infrastructure and host events. Most concerns related to the longevity of the projects in the context of an increasing number of local gardens requiring funding, but some were brought by more altruistic sentiments. For example, Charlie felt guilt about the organisation taking funds from charities that may otherwise be used for tackling serious problems such as child hunger, as well as being paid for one of their roles within the organisation. Despite acknowledging that the particular role was “an awful lot to ask of a volunteer”, Charlie felt conflicted about accepting money generated from charitable inputs. Conversely, many participants were continually seeking funding in order to sustain the garden, but were dissatisfied with the options available. One garden charged a membership fee to generate income, although the coordinator stated that the fees could not cover their expenses and potentially discouraged prospective volunteers.

One coordinator talked of ambitions to fund the garden through operating a household gardening service in order to stop reliance on grants. They recognised, however, that the group was not in an immediate position to start such a venture due to more urgent demands. Another participant talked of the financial success of one of the gardens that ran its own lunchtime café and had regular events, demonstrating that there could be a move towards self-sufficiency. This type of multi-functional model is advocated by Court, Kelly, and Hardman (2022) for increasing financial sustainability. However, despite the apparent resilience, lockdown had greater negative impacts on these types of garden than their grant-funded counterparts, as their money-making activities ceased, leaving them in a more vulnerable position (Schoen et al. 2021).

Three of the groups expressed a desire for kitchen facilities, enabling them to run more community events and feed volunteers. After experiencing “a challenge and a half” in ferrying meals for a public event from another location by bicycle, one coordinator optimistically applied for funding from the National Lottery to build a kitchen.

I was asking him how we could secure a grant for, heading for the bigger fish and asking for £150,000 and the guy was just like, “You’re in no stage to be doing this or asking me, thanks for the phone call, I’ll give you feedback, but just no”.

Similarly, another leader described disappointment at the group having lost use of a church kitchen that they formerly used for Friday night supper, which included garden-grown food. It was a particular blow as the vulnerable group members did not always have access to cooking facilities in their own homes, highlighting how growing food does not necessarily improve food security or the nutritional intakes of target groups (St Clair et al. 2018). While kitchen facilities were desirable, funding opportunities were generally restricted to particular themes which the groups had to adhere to in order to apply, meaning that their expressed needs were unmet by current funding priorities. This emphasis on specific practices by donors can be a barrier to obtaining funding as the complex network of activities that take place in community gardens is difficult to convey (Court, Kelly, and Hardman 2022). One group’s recent grant success was particularly appreciated due to its nature:

That was a good one because that comes fairly unconditionally. That’s yours to spend, you know. Some of the other ones through trusts and charities have been much more specific, based on a business plan and meeting certain criteria and so on.

Most coordinators appeared to be in a continuous cycle of grant seeking to continue activities. As found previously, the continuation of projects after initial grants is usually achieved by the voluntary commitment of only a few individuals (Miller 2015). One participant criticised the competition for funding between similar co-evolving ventures as being unhealthy and unfair, as many deserving groups lacked the capacity to write good grant applications. The cynicism was extended to the whole funding system in which much of the money was perceived to be drained out along the chain, with thousands of hours and pounds spent applying for, and distributing funds, but little of this money actually reaching the gardens themselves. This sentiment echoes the disgruntlement of McClintock (2014) at the whole non-profit industrial complex where public–private ventures put forward as citizen empowerment merely reflect the changing funder priorities, favouring those with the privileges and skills to marshal the funding, resulting in inequalities in social provisioning. The only venture to be able to boast being fully self-sufficient was a school group which used profits from an entrepreneur scheme. The children had learned to print tote bags and make beeswax wraps to sell to school staff and pupils, using the profits to buy their garden materials. This was clearly done within the supportive structure of the school and is likely not a viable option for other groups. Council run or hosted sites were the other groups that had limited incentive to obtain funding, with costs seen as minimal after initial set-up and free supplies of municipal compost available.

### 3.3 Resilience and function through garden model type

A notable aspect of the community groups was that each had their own character, outlook and means of operating depending on how it had evolved and the restraints or capacities of coordinators. Key differences include top-down/ bottom-up approaches, attitudes to inclusion and the affluence of the local neighbourhood. A problem communicated by some coordinators was difficulty in recruiting enough regular volunteers, with a city-wide support worker fearing it may be a problem for the survival of the city’s relatively new community gardens. Interestingly the shortages occurred in Aberdeen’s poorer areas (Scottish Index of Multiple Deprivation (SIMD) deciles 2-5), whereas there was no limitation on the number of volunteers in two of the most affluent areas (SIMD decile 10), which were also the only ones to have paid coordinators. While most other communities shared common features, such as volunteer coordinators who directed other volunteers within set timeframes, an alternative structure was adopted by a residents’ group where the term “community” was viewed very much by its geographical definition (Sharpe, Mair, and Yuen 2016) and all garden contributors were local. This model exhibits features of the “bonding” social capital described by Jackson (2018), which strengthens relationships between neighbours and others of shared demographics already known to each other. When asked about recruiting volunteers, Rowan replied:

You see the problem with volunteers is you have to keep an eye on what they’re doing, and so it becomes another job managing volunteers, whereas if we’re all gardeners doing our own thing then it’s, you know, you’re not responsible.

The residents’ group appeared to have a somewhat anarchist approach to gardening, following a rapid dissolution of its committee when initial meetings soured otherwise amicable relationships. In this case, a model of locals doing what they wanted, when they wanted, was practical and desirable for contributors, successfully demonstrating a resilient structure which is renowned around the city. While this laissez-faire approach caused a degree of contention when the actions of some gardeners clashed with the objectives of others, overall, the prevailing attitude was described as being Zen, acknowledging that contributors were not merely people who met at a weekly gardening session, but neighbours who co-existed on a full-time basis. In this respect the group was truly representative of the local community, a trait that community gardens are often accused of failing to achieve (Furness and Gallaher 2018; Ramsden 2021; St Clair et al. 2018; Traill 2021).

Despite an element of geographical exclusivity, the neighbourhood model welcomed newcomers and was successful at engaging local children on a casual basis. No official activities were run, but around forty children became involved voluntarily by asking if they could help gardeners whom they saw at work. The group facilitated this by buying mini-shovels for them to shovel wood chips and showing them how to pick berries. Children taking food home to their families proved an effective way of engaging the multi-ethnic groups within the neighbourhood, some of which were otherwise seen as being difficult to reach. Further exploration and promotion of “residents’ group“ gardens may therefore help diminish the practice and stereotype of urban gardeners being white, middle-class and unrepresentative of the local community (Furness and Gallaher 2018; St Clair et al. 2018; Traill 2021).

In contrast, another group had an entirely different attitude about who was involved in the community, being more in line with Sharpe, Mair, and Yuen’s (2016) functional community, in transition to a conscious community, with talk of volunteers wanting to form “heart connections“ as much as being engaged in a physical way. While efforts were predominantly aimed within the local community, several student volunteers who lived both locally and around the city were regulars. The coordinator’s attitude to the demographic composition of the community and volunteers involved in the community garden was:

You wouldn’t be a community garden if you didn’t want to raise the cohesion of the community. So, I like to be able to get all sorts of different walks of life involved, … people can then get to understand each other and start to dismantle these stereotypes that people bring up about students or bring up about Mrs Bag-crumb that lives three doors down.

This approach demonstrates the “bridging“ social capital described by Jackson (2018) whereby links are made between people from diverse demographics who would not normally associate with each other. Knowledge exchange is likely to be high within a diverse community and the lead coordinator was indeed keen to encourage skill-building activities and promote safe, efficient work practices. The group appeared to be successful in terms of achievements, engagement and fund gathering, but both coordinators reported fatigue as the biggest challenge, adding that setting up the garden was essentially a full-time job. Activist fatigue is considered to be a unique type of burnout due to the large amount of emotional investment required to understand marginalised communities (Montoya and Chan (2020), with Chen and Gorski (2015) suggesting that social justice activists are especially vulnerable to burnout. Volunteer numbers were reliable at the time of interview, but recent anecdotal evidence suggests there were concerns over the future of the group as the founder had announced their resignation to focus on other aspects of life.

Three other types of structure were observed in the study. In one deprivation zone, a garden was essentially gifted to the local community who appreciated the greenspace and used it for neighbourhood events. However, handing over the considerable responsibilities for physical maintenance was challenging because very few people were interested, and a significant proportion of those were incapable of physical work. The unsuccessful transition may have resulted from the top-down approach that was adopted, which is generally a precarious approach (Court, Kelly, and Hardman 2022; Crossan et al. 2016), although this is difficult to ascertain as a grassroots set-up in another deprivation zone experienced a similar lack of volunteers, while a council-run (top-down) garden in an affluent area had to limit volunteer numbers due to oversubscription. The council-run garden with a paid coordinator demonstrates the attractions of responsibility-free volunteering, which allowed group members to focus on purely hands-on activities. Across the city, volunteers were considered to be in short supply, with the point being raised that the same volunteers turned up in separate places. New gardens therefore inadvertently competed for volunteers already working at other gardens, as volunteers had more options to find a group with their desired ambience. This situation was also identified by a study in Glasgow where, after investigating alternative groups, one volunteer started to view her initial group as “waifs and strays” (Traill 2021). The final structure in Aberdeen was one in which the council allocated polytunnels and ground space to specific groups, usually therapy groups, on a semi-disused council site. For these groups, much of the stressful set-up work was by-passed because the infrastructure was already in place and access rights granted by default.

### 3.4 Future sustainability

The research suggests the future of most of the community gardening activities is dependent on continued funding and other support mechanisms for coordinators to alleviate demands on time and energy. In addition to calls for less reliance on sporadic grant funding for materials and events, several participants wanted secure funding for human resources. Participants from backgrounds as both paid and volunteer coordinators felt the demanding and sometimes full-time nature of the job was more suited to a paid role. There was talk of a city-wide movement to increase volunteer numbers for practical activities and a centralised coordinator to manage them, but that there were not yet solutions on how to achieve it, and that it needed investment for it to happen. The need for more secured coordinator funding was echoed by Billie, whose volunteer organisation was integral for forming connections between growing groups, while what was actually happening was a loss of paid positions. This requirement for a larger source of shared resources such as volunteers, network connections and funding to help smaller movements was also recognised by Gulliver et al. (2023) in the wider environmental movement.

The student group, a teacher, an activist leader and an allotment holder all called for more funding for mentors and trainers. One described the value of a mentor who had taught them about plants, growing and foraging, but whose temporary funding had expired, leaving the group with a perceived lack of skills and confidence. Likewise, another key figure was due to have their funding withdrawn within six months of the interview, leaving an uncertain future for themself and the new communities. Loss of key staff and the resulting lack of clear direction in volunteers has been found to demotivate and risk the loss of volunteers from community projects (Morse et al. 2022; Ramsden 2021), while others have been left fragile after initial funding ran out (Ramsden 2021). The discontinuity of funding for mentors and support workers is an important loss, as these roles link the different communities, which despite the likelihood of competing for the same funding, had a sense of kinship and cooperated in knowledge exchange events and activities. Along with Billie, the mentors were repeatedly mentioned by numerous participants who found them particularly influential and motivational. All school participants also conceded that establishing and running a garden was a big job that needed an enthusiastic teacher to go above and beyond, or support staff for teachers who were not confident with growing skills. “We can create that space [for growing], but we need the expertise to come in to help us and a bit of an injection of ownership and enthusiasm”.

Conversely, the residents’ group were perceptive in their attitude, which welcomed everyone to contribute but was essentially opposed to volunteers or having one or two enthusiastic leaders, seeing that “you’ve not got long-term resilience if somebody moves away”. This would indeed be an issue for all the volunteer led communities, which were in exactly that position of having one or two dedicated leaders, most of whom were balancing paid and voluntary work with personal lives. At the time of writing one group felt in a precarious situation following the resignation of the founder. Such changes are inevitable as people’s lives and interests move on, with the lack of consistency previously being raised as an issue for the survival of groups (Nam and Dempsey 2018). This suggests the need for long-term support and development through continuous capacity building of volunteers (Ramsden 2021) and additional resources dedicated to the continuation of community food groups (Court, Kelly, and Hardman 2022; Nam and Dempsey 2018).

### 3.5 Future directions

The previous lack of research on burnout among volunteers was due to the notion that it is easier for volunteers to resign from their roles without the same consequences as resigning from a stressful paid job (Kulik 2007). However, this assumption was not supported, as garden coordinators holding responsibility felt that their absence could result in the discontinuation of a project or a scheme that they had worked hard to build. Despite the burnout scenario now being well-known amongst environmental activists (Extinction Rebellion 2019), and environmental activism becoming stronger over the past decade, the importance of volunteer leaders in the wider environmental moment has only very recently emerged in the academic literature (Gulliver, Fielding, and Louis 2022; Gulliver et al. 2023). This is a crucial step forward as previous literature does not distinguish between purely “hands-on” volunteers and volunteer coordinators who face considerably different and more demanding challenges.

Kulik (2007) describes volunteering outcomes as binary, resulting in either burnout or satisfaction. What is clear from this study is that the two are not separate, but inextricably linked, with coordinators feeling both love for their work while simultaneously finding it overdemanding. One participant described the experience as “Sometimes it’s overwhelming and I just get fed up with the whole thing, and then other times I get really enthused.” This apparent paradox is somewhat analogous to the argument by McClintock (2014) that urban agriculture is both radical, in its association with grass-roots movements in opposition to the mainstream food system, and neoliberal in that it fits within neoliberal structures to fill the voids in social security provision. Morse et al. (2022) on the other hand, suggested that burnout is dependent on the motivation type, with satisfaction levels affected by whether motivations are self-serving or altruistic, with various authors coming to different conclusions.

The increase in social prescribing of community gardening (Court, Kelly, and Hardman 2022; McGuire, Morris, and Pollard 2022), generates a demand for more policy interventions and secured funding to increase the long-term resilience and sustainability of urban food growing groups. Financial strains evoked by the current cost of living crisis will likely exacerbate time and financial stresses on volunteer coordinators, while funding cuts threaten support workers and future funding. Given that public health interventions deliver a 14: 1 return on investment and that cuts to public health budgets are a false economy (Masters et al. 2017), we agree with Nam and Dempsey (2018) and Court, Kelly, and Hardman (2022) in arguing that wider investment into urban food growing communities is required. This should be focussed on long-term sustainability and centralised support for coordinators. Although capturing the true value of community gardens in terms of public health would be complex, simple measures such as monitoring the sum of volunteer hours accrued in administrative tasks could help inform the value of a paid centralised administrator.

Continued support and funding is particularly important when considering the involvement of those with mental health issues or who are otherwise vulnerable, as such groups are at most risk of harm if projects are terminated (Ramsden 2021). Cessation of funding for therapy groups is particularly detrimental as members usually experience stronger health benefits from gardening than other groups (Soga, Gaston, and Yamaura 2017), with those who were able to continue gardening during lockdown having improved mental health outcomes in relation to the rest of the UK population (Wood, Barton, and Wicks 2022).

The problem with temporary funding leaving participants feeling disappointed, powerless and manipulated is well discussed by St Clair et al. (2018). Further research is needed to assess the survival of the recent waves of community gardens after founding members move on or mentors are lost. Suggested interventions will ideally be based on research that establishes the most constructive organisational model for the circumstances. While opinions in this study support paid roles for centralised mentoring and coordination of volunteers, capacity building and leadership training of a greater number of volunteers may be more beneficial if the former cannot be implemented on a permanent basis. Thus, responsibilities could be shared around the groups, giving reprieve to coordinators and empowering a wider number of volunteers, potentially moving towards self-sustaining systems. We argue that addressing issues relating to long-term community funding and the well-being of volunteer coordinators is not only relevant for community gardens, but is pertinent and transferable to the wider environmental movement and other types of public health interventions.

## 4 Conclusion

Urban food growing and volunteerism both often lead to personal, social and community benefits. This is particularly true for allotmenteers, but those in volunteer coordinator roles face multiple challenges that may negatively affect their own health and the longevity of community projects. Participants in both paid and volunteer roles suggest that coordinator and mentor roles should be remunerated, due to the amount of work and skill involved. However, this would require substantial funding which is problematic due to competition between similar groups, trends in the funding landscape, and unequal opportunities in obtaining funding due to personal skills and social capital. These limitations suggest a need for a funded centralised body that could facilitate coordinators by assisting with tasks such as the coordination of volunteers and the organisation of relevant training and outreach events.

To our knowledge, there has been no targeted research on burnout in community garden volunteers and there appears to be a distinct lack of narratives on the wide-ranging role types of volunteers in this field. Given the undisputed benefits of both gardening and community groups, combined with the increasing tendency towards the social prescribing of individuals into such ventures, and the negative impacts of short-term projects, further research is required into what makes the groups sustainable in the long-term. This would allow policies and funding relating to public health to be channelled in a more effective manner.

## Key policy highlights

- Long-term budgets are required to support the longevity of existing community projects that align with public health goals.
- Funded, centralised bodies are required to help coordinate volunteers and to provide training and empowerment activities to encourage the sharing of workloads.
- Governing bodies need to acknowledge the diverse activities involved in urban food growing and the economic benefits they bring in relation to human, environmental and public health.  NVIVO output of most frequently occurring words (over four letters) in interview transcripts.
